# New evidence for content validity of the German version of the Acute Cystitis Symptom Score: cognitive interview study among patients and experts

**DOI:** 10.1007/s00345-024-05406-2

**Published:** 2025-01-25

**Authors:** Sophie Nestler, Christian Apfelbacher, Ebru Özkan, Kurt Naber, Katharina Piontek

**Affiliations:** 1 Institute of Social Medicine and Health Systems Research, Otto-von-Guericke-University Magdeburg, Medical Faculty, Magdeburg, Germany; 2 Department of Urology, Technical University of Munich, Munich, Germany

**Keywords:** Content validity, Qualitative interviews, COSMIN

## Abstract

**Purpose:**

The Acute Cystitis Symptom Score (ACSS) is a clinically validated instrument to assess symptoms and quality of life in women with uncomplicated urinary tract infections (uUTIs). A previous study examining the content validity of the German version of the ACSS has shown some methodological limitations when rated against the criteria for content validity of the COnsensus-based Standards for the Selection of Health Measurement INstruments (COSMIN) initiative. Extending the existing evidence, the present study aimed to assess the content validity of the German version of the ACSS according to the criteria of the COSMIN methodology.

**Methods:**

In individual cognitive interviews following a structured, standardized interview guide, women with a history of uUTI and experts from different medical fields rated the instructions, items, response options and recall period of the ACSS in terms of relevance, comprehensiveness and comprehensibility. Additionally, the Content Validity Index (CVI) was calculated based on expert assessments to quantify content validity.

**Results:**

A total of 14 patients and 14 experts participated in two rounds of interviews. The overall relevance and comprehensiveness of the instrument were rated as appropriate. Modifications focused on improving comprehensibility. Ten items underwent minor modifications or were given examples to enhance comprehensibility. The scales of five items were linguistically revised. Confirming good content validity, CVI was 0.97.

**Conclusions:**

Comprehensive qualitative assessments support content validity of the ACSS for evaluating symptoms and quality of life in women with uUTIs. Minor modifications addressed comprehensibility. Psychometric validation of the revised ACSS is recommended.

**Supplementary Information:**

The online version contains supplementary material available at 10.1007/s00345-024-05406-2.

## Background

Uncomplicated urinary tract infections (uUTIs) in women are among the most common bacterial infections in primary care [1]. Although the condition is generally self-limiting, antibiotics are commonly used for treatment to achieve rapid symptom resolution [2]. Given the increased rates of resistant uropathogens that may result from potential inappropriate use of antibiotics, alternative approaches, e.g. herbal therapy, are promising [3]. The assessment of the effectiveness of such remedies in clinical trials from the patient’s perspective is considered crucial [4], and growing consensus has emerged about the use of standardized questionnaires (patient-reported outcome measures; PROMs) for this purpose [5].

Initially designed to facilitate initial diagnosis and monitoring of treatment, the Acute Cystitis Symptom Score (ACSS) is a well-established and clinically validated self-reporting questionnaire for use in women with acute uncomplicated cystitis [6–10]. For use as an endpoint measurement tool in clinical trials, high-quality measurement instruments in the domains of validity including content validity and construct validity, reliability and responsiveness are required [11]. A previous systematic review of our research group evaluated the quality of PROMs for use in women with uUTIs according to the guidelines of the COnsensus-based Standards for the Selection of Health Measurement INstruments (COSMIN) group and revealed sufficient content validity, construct validity and responsiveness of the ACSS, emphasizing the potential of the instrument to be used in future research [12, 13]. Content validity is considered the most important measurement property since it is essential that all items of a PROM are relevant, comprehensive, and comprehensible regarding the construct of interest to be measured and the target population [14]. Among the seven content validity studies included in our review, one study examined the content validity of the German version of the ACSS [15], but has some methodological limitations. In particular, patients were solely asked about comprehensibility, but not about relevance and comprehensiveness, and experts were not involved. Moreover, detailed information about different aspects of the procedure were not provided, e.g. concerning the mode of collecting and analyzing qualitative data.

Addressing these limitations, the present study aimed to evaluate the content validity of the German version of the ACSS from the perspective of women with uUTIs and health care professionals (HCPs).

## Methods

### Study design and recruitment of participants

A qualitative interview study following the recommendations of the COSMIN group for the assessment of content validity [13, 16, 17] was performed. The criteria are depicted in Appendix A. Additionally, the process is reported in detail according to the Cognitive Interviewing Reporting Framework (CIRF), a checklist-based guide to create standardized cognitive testing reports [18]. The assessment of content validity according to COSMIN involves a minimum of seven patients from the target group and seven experts from all relevant disciplines. Both patients and experts were recruited via personal contacts and the network of the study team. Female patients at least 18 years old with sufficient German language skills, who had experienced an uUTI within the last three years, were considered eligible for study participation. Efforts were made to achieve demographic diversity regarding age and professional background of the patients. To obtain an interdisciplinary sample of experts, HCPs from various fields including general medicine, urology, gynecology, health and nursing sciences (experts in public health and nursing with focus on research, prevention, patient care, and health promotion across medical, social, and caregiving sciences), as well as research methodology were recruited until the minimum of seven patients and seven experts was reached.

### Material

The ACSS is an 18-item questionnaire comprising of the following four domains:

- Typical symptoms (6 items).
- Differential diagnosis (4 items).
- Quality of life (3 items).
- Additional symptoms (5 items).

The items assessing typical symptoms, differential diagnosis and quality of life are assessed on a 4-point Likert scale ranging from 0 to 3. Additional symptoms are measured on a dichotomous scale (yes/no). Furthermore, the ACSS has a “follow-up” part B to be completed after treatment including the ‘Dynamics’ domain to assess the overall clinical outcome reported by the patient. By summing the scores of each domain, and overall score is calculated. A total score of ≥ 6 points in the ‘Typical symptoms’ domain indicates acute cystitis with high sensitivity and specificity [6]. Developed in Uzbek language, the ACSS was translated into German language according to international guidelines and recommendations including forward and backward translations, revision, correction, cognitive assessment, pilot clinical validation, additional reconciliation and further corrections [15]. The German version of the ACSS is available online: http://www.acss.world/downloads.html.

### Data collection

A standardized interview guide assessing comprehensibility and relevance of the items, response options and recall interval as well as the comprehensiveness of the questionnaire was developed (Appendix B). In cognitive interviews using a combination of the current think aloud methodology and targeted questions (Q-by-Q testing) [19], participants were instructed to read each item aloud, to articulate their thoughts aloud, and to rate comprehensibility and relevance of each item as follows: (1) “item is clear/relevant”, (2) “item needs minor revisions to be clear/relevant”, (3) “item needs major revisions to be clear/relevant”, and (4) “item is not clear/ relevant”. Subsequently, the appropriateness of response options, recall interval, and comprehensiveness of the questionnaire were evaluated using open questions. Prior to the interviews, participants were briefed on the study background, interview procedure and data protection. Interviews were carried out via Zoom video conferencing software by a researcher (SN) who had been trained in conducting qualitative interviews. The interviews were audio-recorded and transcribed verbatim using MaxQDA (MAXQDA 2020.4.2, VERBI Software, 2024). Transcripts were reviewed and anonymized assigning participant identifiers to ensure confidentiality. The interviews lasted about one hour, and a financial incentive was offered to all participants.

### Data coding and analysis

Two independent researchers (SN and EÖ) conducted the coding of the transcripts using Microsoft Excel sheets. Coding was performed for open questions regarding the response options, recall interval, and comprehensiveness. Further, data were re-coded if participants had not responded using the numerical system during the interviews or if the assigned code did not match the participant’s comment.

Potential necessary modifications were discussed within the study team and by consulting the developers of the ACSS if at least three participants did not rate an item as clear or relevant. When discussing modifications, the concrete statements of the participants were considered. Overall, comments of the patients were considered more important concerning the need of potential modifications than comments of the experts. Throughout this process, careful attention was paid to ensure that all modifications aligned with the original version without altering the intended meaning of the items. Where modifications were necessary, the major aim was to improve comprehensibility, relevance and comprehensiveness of the ACSS. This iterative process continued until the questionnaire garnered acceptance from the majority of participants.

Additionally, as quantitative measure of content validity and summary of the assessment of relevance, the Item Content Validity Index (I-CVI) [20], was calculated based on the data from the expert interviews. For this purpose, ratings were dichotomized, with “the item is relevant” receiving 1 point and other ratings receiving 0 points. The total score for each item was divided by the number of experts. A I-CVI of at > 0.78 is considered good [20]. The Scale Content Validity Index/Average (S-CVI/Ave) was calculated by summing all I‐CVI values and dividing by the total number of items. An S‐CVI/Ave value of > 0.90 is considered appropriate [20].

## Results

In total, 14 patients and 14 experts were interviewed in two rounds. The original version of the ACSS was assessed in the first round (31 July to 15 September 2023), and the modified version was evaluated in the second round (14 October to 30 October 2023). Sociodemographic characteristics of the study participants are displayed in Table 1.

**Table 1:** Characteristics of the study participants

	Round 1			Round 2		
	Patients (*N* = 7)	Experts (*N* = 7)	Patients (*N* = 7)		Experts (*N* = 7)	
**Age**,** years** (M, SD)	44.3 ± 21.6	56.6 ± 17.7	24.7 ± 0.9		41.1 ± 11.6	
**Sex** Female, n Male, n	7 0	4 3	7 0		5 2	
**Profession**	5 employees 2 students	3 gynaecologists 2 urologists 2 health scientists	7 students		2 urologists 1 gynaecologist 1 nursing scientist 1 psychologist 1 statistician 1 PRO expert	

Note: M, mean; SD, standard deviation; PRO, patient-reported outcome

### First round of interviews

#### Relevance

All items were considered relevant by patients and experts. However, two experts commented on the inaccuracy of the term “discomfort” used in item 11 (“Please rate how much discomfort you have experienced because of the symptoms in the past 24 hours”), arguing that it describes a general feeling that is not clearly attributable to uUTI. When discussing a potential modification of this items with the developers of the ACSS, it emerged that this item is theoretically assigned to the ‘quality of life’ domain although measuring bothersomeness of symptoms. For a clearer reference to the ‘quality of life’ domain, the wording of item 11 was modified as follows: “Please rate how much the above-mentioned symptoms have affected your overall quality of life in the past 24 hours”. The response options for items 2 (“urgent urination”) to 9 (“discharge from the urethra”), and for item 14 assessing additional symptoms were considered appropriate.

With regard to item 1 (“Frequent urination of small amounts of urine (Going to toilet very often)”), comments primarily pertained to the quantifications in the answer options. Four experts and four patients considered the quantifications beneficial for orientation purposes. In order to avoid significant alterations, it was decided to retain the answer options, but to add “approximately” and “approx. 9–10 times or more” in the fourth response option in order to enable individual assessments.

Regarding the response options for item 10 (“Elevated body temperature/Chills”), three experts preferred a dichotomous scale to assess whether either elevated body temperature or chills are present or not. As this comment was only made by experts and not by patients, the study team decided not to modify the scale. Instead, the study team decided to standardize the item label with focus on body temperature. Accordingly, the term “chills” was replaced by the term “fever” in the item label in order to maintain scalability and standardization.

The 24-hour recall period was deemed appropriate by all participants. Summarizing the relevance ratings from the expert interviews, the S-CVI/Ave value was 0.98, indicating good content validity. Concerning the single items, only item 4 (“Feeling of incomplete bladder emptying”) received a CVI of 0.71, which is reflected in the results of the interviews. Since only two experts questioned the relevance of this item, it was retained as is.

#### Comprehensiveness

Study participants considered the questionnaire appropriate in terms of length, conciseness and completeness. However, some patients noted the need for potential additional questions, such as questions assessing endometriosis, drinking volume, or the suspected cause of the uUTI. Single experts suggested additional questions to assess the amount of fluid consumed, frequency of UTIs per year, pregnancy, urinary incontinence or chronic kidney dysfunction, or sexual history. However, since these suggestions were made by single experts, no modifications have been made.

#### Comprehensibility

The results of the comments on comprehensibility of the items are summarized in Table 2. Certain item formulations such as “involuntary”, “terrible”, or “lumbar region” were not clearly understood by the patients. These comprehension difficulties were predominantly reported by younger patients, indicating potential outdated language. To address this, age-inclusive, easily understandable, and contextually appropriate formulations were selected as modifications. Furthermore, additional formulations were incorporated to enhance comprehensibility for patients from diverse sociodemographic backgrounds. Additional minor modifications were made on item 3 (“Burning pain during urination”), item 5 (“Feeling pain not associated with urination in the lower abdomen”), item 6 (“Blood seen in urine”) and item 14 (“Additional symptoms”) with single additions for the sake of consistency by the developers of the ACSS, even though they did not require urgent changes based on the results from the interviews.

**Table 2:** Comprehensibility issues

			Patients (*n*)	Examples	Modification
**Item**					
2	“*Strong*,* involuntary* urge to urination”		3	Patient is not sure, what “involuntary” means in the context.	Reformulation of the words “strong” and “involuntary”.
7	“Pain in the *lumbar region (flank*) *often unilateral (on one side)”		3	Patient is not sure what is meant by the lumbar region and cannot clearly localize the pain based on this description.	Additional description of the lumbar region by “lower lateral back pain”.
8	“*New* or increasing vaginal discharge”		3	Patient is not sure what new discharge means in the context.	Reformulation of the term “new” and addition of a description of the discharge in terms of “quantity”, appearance and/or odour”.
14	… “*Diabetes*?”		3	Patient is not sure what is meant by “Diabetes” and could only suspect diabetes.	Inclusion of the term “diabetes mellitus” for consistency and understanding.
**Response options**					
11		0 No discomfort (No symptoms at all. I feel as good as usual.) 1 Mild discomfort (I feel a little worse than usual.) 2 Moderate discomfort (I feel much worse than usual.) 3 Severe discomfort (I feel terrible.)	4	Patient perceived formulations such as “discomfort” or “feeling terrible” as unclear or informal.	Reformulation of the response option using a less informal term.
12/ 13		0 Not interfered at all (working as usual, without symptoms) 1 Mildly interfered (I work a little less because of the symptoms) 2 Moderately interfered (everyday work has become strenuous) 3 Severely interfered (I am practically unable to work)	4	Patient perceived formulations such as “working” as unclear or informal.	The explanations of the response options have been revised by replacing the term “working” with “activity” and using a less informal term.

Since modifications of a questionnaire require a new round of content validation interviews in accordance with the COSMIN guidelines, the revised version was evaluated in a second round of interviews involving another seven patients and seven experts.

### Second round of interviews

#### Relevance

The response options for item 1 (“Frequent urination of small amounts of urine (Going to toilet very often)”) and 10 (“Elevated body temperature/fever”) as discussed in the first round of interviews were specifically evaluated. The majority of experts (*n* = 6) and one patient advocated for removing the quantifications included in the response options for item 1. Although patient perspectives were prioritized throughout the study, the feedback from the majority of experts (6 out of 7) was considered particularly valuable in this case. Experts highlighted that fixed frequency options may not accurately capture individual variations in urination patterns, and emphasized the importance of the subjective sensation. Following their recommendation, the quantification of frequency was removed. Additional minor modifications in the wording of the response options of item 1 were performed in consultation with the developers of the ACSS to maintain homogeneity of the wording. The scale of item 10 was suggested to be dichotomized by two experts and one patient. Since this suggestion was made by only one participant, it was decided not to modify the scale based on the decision of the first interview round.

Concerning item 9 (“Discharge of the urethra”), it was noted that distinguishing between “abnormal vaginal discharge” and “discharge of the urethra” is difficult, and the relevance of this item was questioned by two experts and one patient. However, the study team decided to retain this item since only one woman doubted its relevance, and since itis primarily important for differential diagnosis. Minor changes in wording of item 15 (“Dynamics”) were also made to improve homogeneity in the formulations.

Summarizing the expert interviews’ relevance assessments resulted in a CVI/Ave value of 0.97, indicating good content validity. The results of the first round of interviews on item 4 (“Feeling of incomplete bladder emptying”) were also occasionally reflected in the results of the second round, resulting in an I-CVI of 0.71 not requiring modification. Furthermore, the comments on item 9 (“Discharge of the urethra”) are reflected in an I-CVI of 0.86.

#### Comprehensiveness

The compactness of the ACSS was also noted positively in the second round of interviews. Only individual respondents made comments on the questionnaire and suggested to include questions about the frequency of bladder infections per year, suspected causes of uUTI and medication use. In addition, open questions on other symptoms or impairments were suggested by individual respondents. As these are individual opinions, no questions were added.

#### Comprehensibility

There were no problems with the modified version of the ACSS resulting from the first round of interviews in terms of comprehensibility. However, item 12 (“Please indicate how these symptoms have interfered with your everyday activities/work in the past 24 hours”), was slightly modified by adding examples to better differentiate it from item 13 (“Please indicate how these symptoms have interfered with your social activities in the past 24 hours”). These suggested additions were noted by two patients. As this is not a change to the wording of the item, but merely the addition of examples, this change has been complied with. These additional examples contribute to better comprehensibility for patients. The modified and the final version of the ACSS are depicted in Appendix C and D, respectively.

## Discussion

Both patients and experts rated the overall relevance and comprehensiveness of the ACSS as appropriate. Modifications were primarily made to improve comprehensibility. Ten items underwent minor modifications or were given examples to enhance comprehensibility, and the scales of five items were linguistically revised.

Strengths of the present study encompass the adherence to the recommendations of the COSMIN group, which represent international, research-based practice in measurement and statistics for instruments in health care, thereby supporting the development of high-quality measurement instruments [21]. Further, the results are reported following the CIRF which provides a structured methodology for reporting how cognitive testing was conducted to ensure transparency by clearly outlining the procedures and rationale behind cognitive interview methods [18]. The ACSS was evaluated from a variety of perspectives based on comprehensive qualitative assessments involving women of different ages, HCPs from various fields of medicine and experts in research methodology. Additional quantitative analyses provided support for the findings obtained from qualitative interviews. A limitation may arise from the fact that the group of patients comprised primarily younger women with higher education and homogeneous cultural background. To ensure the instrument’s comprehensibility and applicability across diverse populations, future research should assess content validity of the ACSS in populations with a wider range of cultural backgrounds, ages, and educational levels.

Overall, our findings indicate that the ACSS is a suitable tool for the measurement of the intended constructs. Notably, while the ACSS is hypothesized to measure typical and differential symptoms, quality of life and additional symptoms, the underlying measurement model has not been analyzed yet. In this regard, independent from the interview data, an important methodological limitation of the ACSS emerged when discussing the content of the questionnaire with the developers. Item 11, which is theoretically assigned to the ‘quality of life’ domain, was considered as problematic since it is measuring bothersomeness of symptoms and not impact of symptoms on quality of life. Subsequently, this item was modified by explicitly asking for the impact of symptoms on the overall quality of life. In view of these issues, validation studies examining the factor structure of the ACSS are highly warranted.

## Conclusion

Based on comprehensive qualitative assessments following the standards of the COSMIN group, the present study provided high-quality data on the content validity of the German version of the ACSS. Overall, the findings indicate that the ACSS shows high content validity for the assessment of symptoms and quality of life in women with uUTIs. Modifications of the instrument concerned comprehensibility issues. Future studies should focus on investigating the psychometric properties of the modified ACSS, particularly its structural validity and reliability in terms of internal consistency of the scales.

## Electronic Supplementary Material

Below is the link to the electronic supplementary material.

Supplementary Material 1

Supplementary Material 2

## Data Availability

The data used and/or analysed during the current study are available from the corresponding author on reasonable request.

## Abbreviations

ACSS: Acute Cystitis Symptom Score
CIRF: Cognitive Interviewing Reporting Framework
COSMIN: COnsensus-based Standards for the Selection of Health Measurement Instruments
I-CVI: Item Content Validity Index
uUTI: uncomplicated urinary tract infection
