# Supplementary material for: New evidence for content validity of the German version of the Acute Cystitis Symptom Score: cognitive interview study among patients and experts
Source: World J Urol. 2025 Jan 25;43(1):86. doi: 10.1007/s00345-024-05406-2 (PMC11909055; doi:10.1007/s00345-024-05406-2)
Supplement: Supplementary file 1 — Supplementary Material 1 [file 345_2024_5406_MOESM1_ESM.docx]

Appendix A: COSMIN criteria for the evaluation of the quality of studies on content validity

Criteria for “very good ratings” are depicted.

| **No** | **Design requirements** | **Requirement** | **Details** |
| --- | --- | --- | --- |
| 1a | Was an appropriate method used to ask patients and experts whether each item is relevant for the construct of interest? | Widely recognized or well justified method used | The interview guide contained general questions whether each item is relevant for the construct of interest. |
| 1b | Was an appropriate method used for assessing the comprehensiveness of the PROM? |  | The interview guide contained general questions about the overall instrument and missing concepts. |
| 1c | Was an appropriate qualitative method used for assessing the comprehensibility of the PROM instructions, items, response options, and recall period? |  | The interview guide contained general questions about comprehensibility of the PROM instructions, items, response options, and recall period. |
| 2 | Was each item tested in an appropriate number of patients and experts? | For qualitative Studies ≥ 7 | Each item was tested by 7 patients and 7 experts in each round of interviews. |
| 3 | Were professionals from all relevant disciplines included? | Professionals from all required disciplines were included | Experts from various fields including general medicine, urology, gynecology, health and nursing sciences, psychology, and research methodology were interviewed, and their comments were confidently documented. |
| 4 | Were skilled group moderators/interviewers used? | Skilled group moderators/ interviewers used | All interviews were conducted by a trained and experienced interviewer (SN). |
| 5 | Were the group meetings or interviews based on an appropriate topic or interview guide? | Appropriate topic or interview guide | The interview guide was based on five criteria for relevance, one for comprehensiveness, and four for comprehensibility using a coding system. |
| 6 | Were the group meetings or interviews recorded and transcribed verbatim? | All group meetings or interviews were recorded and transcribed verbatim | Each interview was audio-recorded using the record function of Zoom video conferencing software. Audio data were transcribed verbatim. |
| 7 | Was an appropriate approach used to analyze the data? | A widely recognized or well justified approach was used | The analyses followed a coding guideline. |
| 8 | Were at least two researchers involved in the analysis? | At least two researchers involved in the analysis | EÖ and SN independently coded the data. The results were analyzed by EÖ and SN and subsequently discussed within the entire study team. |

Appendix B: Cognitive interview guide

**Welcoming**

Thank you for taking the time for the interview. With the interview, you are supporting a research project of the Institute for Social Medicine and Health Systems Research. In this project, we aim to evaluate a questionnaire assessing symptoms and impact of acute uncomplicated urinary tract infections in women. For this purpose, we would like to discuss with you whether the items are relevant and comprehensive. We have invited you for this interview since you have experienced an uncomplicated urinary tract infection in the past, and your experiences are a valuable contribution to the evaluation and potential modification of the questionnaire. The interview will probably last for about 60 minutes.

**Instruction**

Before we start, it is important for me to mention that there are no right or wrong answers. For this interview, your opinion and your experiences are crucial. I will lead the conversation. We will evaluate the questionnaire item-by-item using the criteria I have sent to you. Please open the file to you have it in front of you all the time. Alternatively, you can write the criteria down or print the file out.

| **Comprehensibility** | |  | **Relevance** | |
| --- | --- | --- | --- | --- |
| **Code** | **Meaning** |  | **Code** | **Meaning** |
| 1 | Wording is clear |  | 1 | Activity is relevant |
| 2 | Must be presented slightly different |  | 2 | Must be presented slightly different |
| 3 | Must be presented clearly different |  | 3 | Must be presented clearly different |
| 4 | Wording is not clear |  | 4 | Wording is not clear |

For each item, we will assess the comprehensibility of the wording and the relevance of its content. Please indicate whether the item is clearly understandable for you or whether you consider minor or major changes necessary to understand the item better. Likewise, we will evaluate the relevance of each item.

We would like to record the interview for later analysis. Your data will be stored and analyzed anonymously with no linkage to your person. If you need a break during the interview, just let me know anytime. If you agree, I would start the record now.

**Assessment**

Note: In addition to the evaluation of the questionnaire according to the predefined criteria, participants are welcome to make suggestions for alternative wording or content regarding the instructions, items, response options and recall period.

*General instruction:*

At the beginning, I would like to ask you to read the instruction for the questionnaire aloud.

- How clear is the instruction for you?
- Are there any sentences that you consider not relevant?

*Comprehensibility (item-by-item):*

- Please read the sentence aloud. With regard to the response notions noted for comprehensibility and clarity, how would you rate this item?

*Relevance (item-by-item):*

- How relevant is this item for you? Here, too, you may use the defined response options.

*Recall period und response options (assessed after the evaluation of all items):*

- In retrospect, all questions refer to the last 24 hours. Do you consider this recall period appropriate?
- Let's take a closer look at the response options. Do you consider the response options appropriate and understandable?

*Overall impression:*

- What is your overall impression of the questionnaire?
- Are there important aspects you think that are missing in the questionnaire?
- Do you have any suggestions for improving the questionnaire?

*Summary:*

- Is there anything else you would like to add to our conversation? Perhaps an aspect that has not been mentioned so far, but you consider important?
- The interview is now finished. Thank you very much for taking the time to meet with me and I wish you all the best.
