# Supplementary material for: New evidence for content validity of the German version of the Acute Cystitis Symptom Score: cognitive interview study among patients and experts
Source: World J Urol. 2025 Jan 25;43(1):86. doi: 10.1007/s00345-024-05406-2 (PMC11909055; doi:10.1007/s00345-024-05406-2)
Supplement: Supplementary file 2 — Supplementary Material 2 [file 345_2024_5406_MOESM2_ESM.docx]

**Appendix C: Modified version of the Acute Cystitis Symptom Score (ACSS) – English version**

***Note: For information purposes only, not linguistically validated***

| **Please indicate whether you have had the following symptoms during the past 24 hours, and how severe they were: (Please mark only one answer for each symptom)** | | | | | |
| --- | --- | --- | --- | --- | --- |
| **Typical Symptoms** | | | | | |
|  |  | **0** | **1** | **2** | **3** |
| 1 | Frequent urination of small amounts of urine *(going to the toilet very often)* | 🞎  No, normal | 🞎  Yes, slightly more often  than usual | 🞎  Yes, noticeably more often than usual | 🞎  Yes, very  frequent |
|  |  | **None** | **Yes, mild** | **Yes, moderate** | **Yes, severe** |
| 2 | Sudden, uncontrollable urge to urinate | 🞎 | 🞎 | 🞎 | 🞎 |
| 3 | Burning pain when urinating | 🞎 | 🞎 | 🞎 | 🞎 |
| 4 | Feeling incomplete bladder emptying | 🞎 | 🞎 | 🞎 | 🞎 |
| 5 | Feeling pain in the lower abdomen *(below the belly button)* | 🞎 | 🞎 | 🞎 | 🞎 |
| 6 | **Blood seen in urine *(without menses)*** | 🞎 | 🞎 | 🞎 | 🞎 |
| **Differential** | | | | | |
| 7 | Pain in the lumbar region *(Flank, lateral, lower back area)** | 🞎 | 🞎 | 🞎 | 🞎 |
| 8 | Abnormal vaginal discharge *(abnormal amount, color and/or odor)* | 🞎 | 🞎 | 🞎 | 🞎 |
| 9 | Purulent discharge from the urinary opening *(without urination)* | 🞎 | 🞎 | 🞎 | 🞎 |
| 10 | a) Feeling high body temperature (above 37,5°C) /fever | 🞎 | 🞎 | 🞎 | 🞎 |
|  | b) Have you measured the temperature?    🞎 No 🞎 Yes | 🞎  ≤ 37,5°C | 🞎  37,6-37,9°C | 🞎  38,0-38,9°C | 🞎  ≥ 39,0°C |

*** *often only on one side*

| **Quality of Life** | | | | | | |
| --- | --- | --- | --- | --- | --- | --- |
| 11 | | **Please rate how much the above-mentioned symptoms have interfered with your quality of life within the past 24 hours *(Please mark the most appropriate answer)*:** | | | | |
|  |  | 🞎 0 | | Did not interfere at all *(No symptoms at all. I feel as good as usual)* | | |
|  |  | 🞎 1 | | Mildly interfered *(I feel a little worse than usual)* | | |
|  |  | 🞎 2 | | Moderately interfered *(I feel much worse than usual)* | | |
|  |  | 🞎 3 | | Severely interfered *(I feel very bad)* | | |
|  | | | | | | |
| 12 | | **Please indicate how these symptoms have interfered with your everyday activities/work *(e.g. work, housework, shopping, etc.)* within the last 24 hours. Please select the most appropriate answer).** | | | | |
|  |  | 🞎 0 | | Did not interfere at all (*My activities are as usual*) | | |
|  |  | 🞎 1 | | Mildly interfered *(My activities are not very restricted)* | | |
|  |  | 🞎 2 | | Moderately interfered *(My activities are significantly restricted)* | | |
|  |  | 🞎 3 | | Severely interfered *(My activities are very much restricted)* | | |
|  | | | | | | |
| 13 | | **Please indicate how these symptoms have interfered with your social activities (*e.g. visiting people, meeting with friends, etc.)* within the past 24 hours. Please mark the most appropriate answer.** | | | | |
|  |  | 🞎 0 | | Did not interfere at all (*My activities are as usual)* | | |
|  |  | 🞎 1 | | Mildly interfered (*My activities are not very restricted)* | | |
|  |  | 🞎 2 | | Moderately interfered *(My activities are significantly restricted)* | | |
|  |  | 🞎 3 | | Severely interfered *(My activities are very much restricted)* | | |
|  | | | | | | |
| **Additional** | | | | | | |
| 14 | | **Please indicate whether you have the following at the time of completion of this questionnaire:** | | | | |
|  |  |  | | | **No** | **Yes** |
|  |  | Menstruation (*menses period*)? | | | 🞎 | 🞎 |
|  |  | *Premenstrual syndrome (PMS) - Symptoms before the menses/menstrual period?* | | | 🞎 | 🞎 |
|  |  | Climacteric syndrome (symptoms during the menopause, e.g. hot flushes)? | | | 🞎 | 🞎 |
|  |  | Known pregnancy? | | | 🞎 | 🞎 |
|  |  | Known diabetes mellitus *(high blood sugar)?* | | | 🞎 | 🞎 |
|  | | | | | | |
| **Dynamik** | | | | | | |
|  | **Please indicate whether your symptoms have changed since the first part of the questionnaire (Please mark the most appropriate answer).** | | | | | |
|  | 🞎 0 | | I now feel symptom-free again *(All symptoms are completely gone)* | | | |
|  | 🞎 1 | | I feel much better *(Most of the symptoms are gone)* | | | |
|  | 🞎 2 | | I feel somewhat better *(Most of the symptoms are still there)* | | | |
|  | 🞎 3 | | There are barely any changes *(I still have about the same symptoms)* | | | |
|  | 🞎 4 | | I feel worse *(My condition has become worse)* | | | |

Appendix D: Final German version of the Acute Cystitis Symptom Score (ACSS)

| **Bitte geben Sie an, ob Sie die unten genannte Symptome innerhalb der letzten 24 Stunden bemerkt haben, und bewerten Sie bitte deren Intensität (*nur eine Antwort für jedes einzelne Symptom).*** | | | | | |
| --- | --- | --- | --- | --- | --- |
| **Typische Symptome** | | | | | |
|  |  | **0** | **1** | **2** | **3** |
| 1 | Häufiges Wasserlassen mit geringen Urinportionen (*wiederholte WC-Besuche*) | 🞎  Nein, normal | 🞎  Ja, etwas häufiger als sonst | 🞎  Ja, merklich häufiger als sonst | 🞎  Ja, sehr häufig |
|  |  | **Nein** | **Ja, wenig** | **Ja, mäßig** | **Ja, stark** |
| 2 | Plötzlicher, unkontrollierbarer Harndrang | 🞎 | 🞎 | 🞎 | 🞎 |
| 3 | Brennende Schmerzen beim Wasserlassen | 🞎 | 🞎 | 🞎 | 🞎 |
| 4 | Gefühl einer unvollständigen Harnblasenentleerung | 🞎 | 🞎 | 🞎 | 🞎 |
| 5 | Schmerzen *(unangenehmes Druckgefühl)* im Unterbauch (unterhalb des Nabels) | 🞎 | 🞎 | 🞎 | 🞎 |
| 6 | **Sichtbares Blut im Urin (außerhalb der Regel/Periode)** | 🞎 | 🞎 | 🞎 | 🞎 |
| **Differenzialdiagnose** | | | | | |
| 7 | Schmerzen in der Lendengegend *(Flanke, seitlicher, unterer Rückenbereich)** | 🞎 | 🞎 | 🞎 | 🞎 |
| 8 | Ungewöhnlicher Ausfluss aus der Scheide (Menge, Farbe und/oder Geruch) | 🞎 | 🞎 | 🞎 | 🞎 |
| 9 | Eitriger Ausfluss aus der Harnröhre *(unabhängig vom Wasserlassen)* | 🞎 | 🞎 | 🞎 | 🞎 |
| 10 | 1. Erhöhte Körpertemperatur (über 37,5°C) / Fieber | 🞎 | 🞎 | 🞎 | 🞎 |
|  | 1. Haben Sie die Temperatur gemessen?   🞎 Nein 🞎 Ja | 🞎  ≤ 37,5°C | 🞎  37,6-37,9°C | 🞎  38,0-38,9°C | 🞎  ≥ 39,0°C |

**oft nur auf einer Seite*

| **Lebensqualität** | | | | | | |
| --- | --- | --- | --- | --- | --- | --- |
| 11 | | **Bitte geben Sie an, wie stark die oben genannten Symptome Ihre Lebensqualität insgesamt innerhalb der letzten 24 Stunden beeinträchtigt haben. Wählen Sie bitte die am ehesten zutreffende Antwort).** | | | | |
|  | | 🞎 0 | | Überhaupt nicht beeinträchtigt *(Ich fühle mich so gut wie immer)* | | |
|  | | 🞎 1 | | Ein wenig beeinträchtigt *(Ich fühle mich etwas schlechter als sonst)* | | |
|  | | 🞎 2 | | Bedeutend beeinträchtigt *(Ich fühle mich deutlich schlechter als sonst)* | | |
|  | | 🞎 3 | | Stark beeinträchtigt *(Ich fühle mich sehr schlecht)* | | |
|  | | | | | | |
| 12 | | **Bitte geben Sie an, wie weit die oben genannten Symptome Ihre alltägliche Aktivität / Leistungsfähigkeit innerhalb der letzten 24 Stunden beeinträchtigt haben. Wählen Sie bitte die am ehesten zutreffende Antwort).** | | | | |
|  | | 🞎 0 | | Überhaupt nicht beeinträchtigt *(Meine Aktivitäten sind wie immer)* | | |
|  | | 🞎 1 | | Ein wenig beeinträchtigt *(Meine Aktivitäten sind wenig eingeschränkt)* | | |
|  | | 🞎 2 | | Bedeutend beeinträchtigt *(Meine Aktivitäten sind deutlich eingeschränkt)* | | |
|  | | 🞎 3 | | Stark beeinträchtigt *(Meine Aktivitäten sind sehr eingeschränkt)* | | |
|  | | | | | | |
| 13 | | **Bitte geben Sie an, wie weit die oben genannten Symptome Ihre gesellschaftlichen Aktivitäten (Besuche machen, sich mit Freunden treffen usw.) innerhalb der letzten 24 Stunden beeinträchtigt haben. Wählen Sie bitte die am ehesten zutreffende Antwort).** | | | | |
|  | | 🞎 0 | | Überhaupt nicht beeinträchtigt *(Meine Aktivitäten sind wie immer)* | | |
|  | | 🞎 1 | | Ein wenig beeinträchtigt *(Meine Aktivitäten sind wenig eingeschränkt)* | | |
|  | | 🞎 2 | | Deutlich beeinträchtigt *(Meine Aktivitäten sind deutlich eingeschränkt)* | | |
|  | | 🞎 3 | | Stark beeinträchtigt *(Meine Aktivitäten sind sehr eingeschränkt)* | | |
|  | | | | | | |
| **Begleitumstände** | | | | | | |
| 14 | | **Bitte geben Sie an, ob zum Zeitpunkt des Ausfüllens des Fragenbogens bei Ihnen folgendes zutrifft:** | | | | |
|  | |  | | | **Nein** | **Ja** |
|  | | Menstruation *(Regel/Periode)*? | | | 🞎 | 🞎 |
|  | | Prämenstruelle Beschwerden *(Beschwerden in der Zeit vor der Regel/Periode)*? | | | 🞎 | 🞎 |
|  | | Klimakterisches Syndrom *(Beschwerden in den Wechseljahren,  z.B. Hitzewallungen)*? | | | 🞎 | 🞎 |
|  | | Schwangerschaft bekannt? | | | 🞎 | 🞎 |
|  | | Diabetes mellitus *(Zuckerkrankheit)* bekannt? | | | 🞎 | 🞎 |
|  | | | | | | |
| **Dynamik** | | | | | | |
|  | Bitte geben Sie an, ob sich Ihre Symptome seit dem ersten Teil des Fragebogens, verändert haben (Bitte kreuzen Sie die am ehesten zutreffende Antwort an). | | | | | |
|  | 🞎 0 | | Ich fühle mich jetzt wieder beschwerdefrei *(Alle Symptome sind endgültig vergangen)* | | | |
|  | 🞎 1 | | Mir geht es jetzt wesentlich besser *(Die Mehrheit der Symptome ist vergangen)* | | | |
|  | 🞎 2 | | Ich fühle mich jetzt nur gering besser *(Die Mehrheit der Symptome ist immer noch da)* | | | |
|  | 🞎 3 | | Es gibt keine Änderung meines Zustandes *(Alle Symptome sind noch vorhanden)* | | | |
|  | 🞎 4 | | Es ist jetzt schlimmer geworden *(Mein Zustand hat sich verschlechtert)* | | | |
